# One trigger, two systems: post-infectious severe Guillain-Barre syndrome with concurrent central nervous system demyelination”: a case report

**DOI:** 10.1093/omcr/omag202

**Published:** 2026-10-25

**Authors:** Mohammad Numan, Mohammad Alhatou, Zaeem Hussain, Maidah Hussain, Abraham Alhatou, Wasfy Hamad

**Affiliations:** Department of Pulmonary and MICU, Al-Khor Hospital, Hamad Medical corporation, Zone 74, Um Enaig street, Al Thakhera, P.O. Box 3050 Doha, Qatar; Internal Medicine, Collage of Medicine, Qatar University, Zone 68, Al- Jameaa street, Jelaiah, P.O. Box 2713, Doha, Qatar; Department of Neurology, Al-Khor Hospital, Hamad Medical corporation, Zone 74, Um Enaig street, Al Thakhera, P.O. Box 3050 Doha, Qatar; Internal Medicine, Collage of Medicine, Qatar University, Zone 68, Al- Jameaa street, Jelaiah, P.O. Box 2713, Doha, Qatar; Karachi Institute of Medical Sciences, CMH Malir, Karachi, Sindh 75080, Pakistan; Lincoln Memorial University – DeBusk College of Osteopathic Medicine (LMU-DCOM), Knoxville, 9737 Cogdill Road, TN 37932, United States; Department of Pulmonary and MICU, Al-Khor Hospital, Hamad Medical corporation, Zone 74, Um Enaig street, Al Thakhera, P.O. Box 3050 Doha, Qatar

**Keywords:** acute demyelinating polyradiculoneuropathy, central nervous system demyelinating disease, post infectious demyelinating disease, autonomic dysfunction

## Abstract

Combined central and peripheral demyelination is a rare but serious neurological condition that is associated with poor outcomes. We report a 31-year-old male presenting with rapidly progressive Acute inflammatory demyelinating polyradiculoneuropathy (AIDP) variant of Guillain–Barré syndrome following an upper respiratory tract infection. The course was complicated by respiratory failure requiring intubation and significant autonomic dysfunction. Despite treatment with intravenous immunoglobulin, there was minimal clinical improvement. Further imaging of the central nervous system revealed multiple acute demyelinating lesions involving the brain and spinal cord. The patient subsequently received plasmapheresis and high-dose intravenous steroids without significant neurological recovery. A severe hospital-acquired infection delayed rituximab initiation until four months after symptom onset; however, neurological recovery remained minimal. This case highlights the rare coexistence of post-infectious central and peripheral demyelination. Early recognition of central involvement in patients with poor response to standard GBS therapy may help guide escalation of immunotherapy and potentially improve outcomes.

## Introduction

Guillain-Barré syndrome (GBS) is an autoimmune polyradiculoneuropathy that is usually triggered by an infection and mediated by mechanisms such as molecular mimicry usually after an infection and has a median annual incidence of 1.3 cases per population of 100 000 [1]. GBS can have many types based on its pathology and symptoms. Acute inflammatory demyelinating polyradiculoneuropathy (AIDP), the most common variant, is characterized by macrophage- and T-cell–mediated demyelination of peripheral nerves and nerve roots, leading to conduction block and, in severe cases, secondary axonal degeneration [2].

Although GBS is typically a disease of the peripheral nervous system, there have been some cases reported where concurrent CNS involvement has been seen. This overlap challenges the traditional distinction between central and peripheral demyelinating diseases and has led to the concept of combined central and peripheral demyelination (CCPD). Conditions such as Acute disseminated encephalomyelitis, Multiple sclerosis, and Myelin oligodendrocyte glycoprotein antibody-associated disease may share overlapping clinical and radiological features with these syndromes, particularly in the context of post-infectious immune activation [3].

The coexistence of GBS with extensive central nervous system demyelination is exceedingly rare. In this report, we describe a case of severe post-infectious AIDP with secondary axonal degeneration, concurrent central nervous system demyelination, and life-threatening autonomic dysfunction, highlighting the expanding spectrum of neuroimmunological overlap syndromes.

## Case presentation

A previously healthy 31-year-old man, fully independent in all daily activities, presented to our hospital with a 1-day history of rapidly progressive ascending weakness that began in the lower limbs and quickly involved all four extremities. He also reported generalized pain affecting the neck, jaw, and limbs. Ten days prior to presentation, he had experienced fever, sore throat, cough, and diarrhea, which resolved spontaneously.

Within 24 hours of admission, the weakness progressed to severe flaccid quadriplegia with marked neurological deterioration, including respiratory muscle involvement, areflexia, urinary retention, and early bulbar symptoms, necessitating urgent intubation and admission to the intensive care unit for ventilatory support.

There was no prior history of neurological disease, chronic illness, recent vaccination, or significant medication use. Family and psychosocial histories were unremarkable, with no known genetic predisposition to neurological disorders.

Upon admission to the intensive care unit, the patient was conscious and oriented. Neurological examination showed severe bulbar weakness and bilateral facial diplegia involving both the upper and lower facial muscles. The remaining cranial nerve examination was unremarkable. He had flaccid tetraplegia with Medical Research Council (MRC) grade 0/5 power in all extremities, while muscle bulk was preserved. Sensory examination demonstrated global hypoesthesia below the C4 level, with more prominent impairment of spinothalamic modalities (pain and temperature sensation). Deep tendon reflexes were absent throughout, and plantar responses were mute bilaterally. Examination of the other systems was unremarkable.

Initial laboratory investigations, including complete blood count, kidney function tests, liver function tests, and serum electrolytes, were within normal limits. However, C-reactive protein was mildly elevated at 14 mg/l (normal range: 0–5 mg/l).

Cerebrospinal fluid (CSF) analysis on admission demonstrated pleocytosis with 32 cells/μl (reference range: 0–5 cells/μl), of which 81% were lymphocytes. CSF protein of 0.46 g/l (reference range: 0.15–0.45 g/l), and the CSF-to-serum glucose ratio was 57% (normal > 60%). CSF polymerase chain reaction (PCR) testing was negative for cytomegalovirus, human herpesvirus 6, herpes simplex virus types 1 and 2, varicella-zoster virus, parechovirus, enterovirus, *Mycobacterium tuberculosis*, and *Cryptococcus neoformans*. CSF cultures were negative.

Repeat CSF analysis 10 days after admission showed persistent pleocytosis with 42 cells/μl, including 46% neutrophils and 35% lymphocytes. CSF protein was markedly elevated at 4.18 g/l. Further CSF studies demonstrated elevated immunoglobulin G (IgG) at 1613 mg/l (normal range: 0–34 mg/l) and an increased IgG index of 0.9 (reference range: 0.3–0.6). Matching oligoclonal bands were identified in both CSF and serum. Serological testing for human immunodeficiency virus (HIV), hepatitis B, and hepatitis C was negative. Rheumatological workup revealed a low titer positive antinuclear antibody (ANA) test (1:40), while extractable nuclear antigen (ENA) panel testing was negative.

Further investigations included serum protein electrophoresis with immunofixation, which revealed no monoclonal bands. Anti–myelin oligodendrocyte glycoprotein (MOG) and anti–aquaporin-4 (AQP4) antibodies were non-reactive. Testing for nodal/paranodal antibodies, including anti-neurofascin-155, anti-contactin-1, and anti-CASPR1 antibodies, was not performed, as these investigations were unavailable at our institution.

Nerve conduction studies showed a severe motor axonal neuropathy with conduction block (>85% in the right ulnar nerve across the forearm; > 95% in both tibial nerves when stimulated at the popliteal fossa). Sensory studies demonstrated markedly reduced bilateral median and ulnar SNAP amplitudes, with preservation of sural, radial, and superficial peroneal responses bilaterally. Although conduction block at the popliteal fossa is often considered technically unreliable, its presence—when marked, reproducible, and accompanied by supportive features such as sural sparing—may still favor a demyelinating process, supporting a diagnosis of AIDP over Acute Motor and Sensory Axonal Neuropathy (AMSAN) (Figure 1).

**Figure 1:**
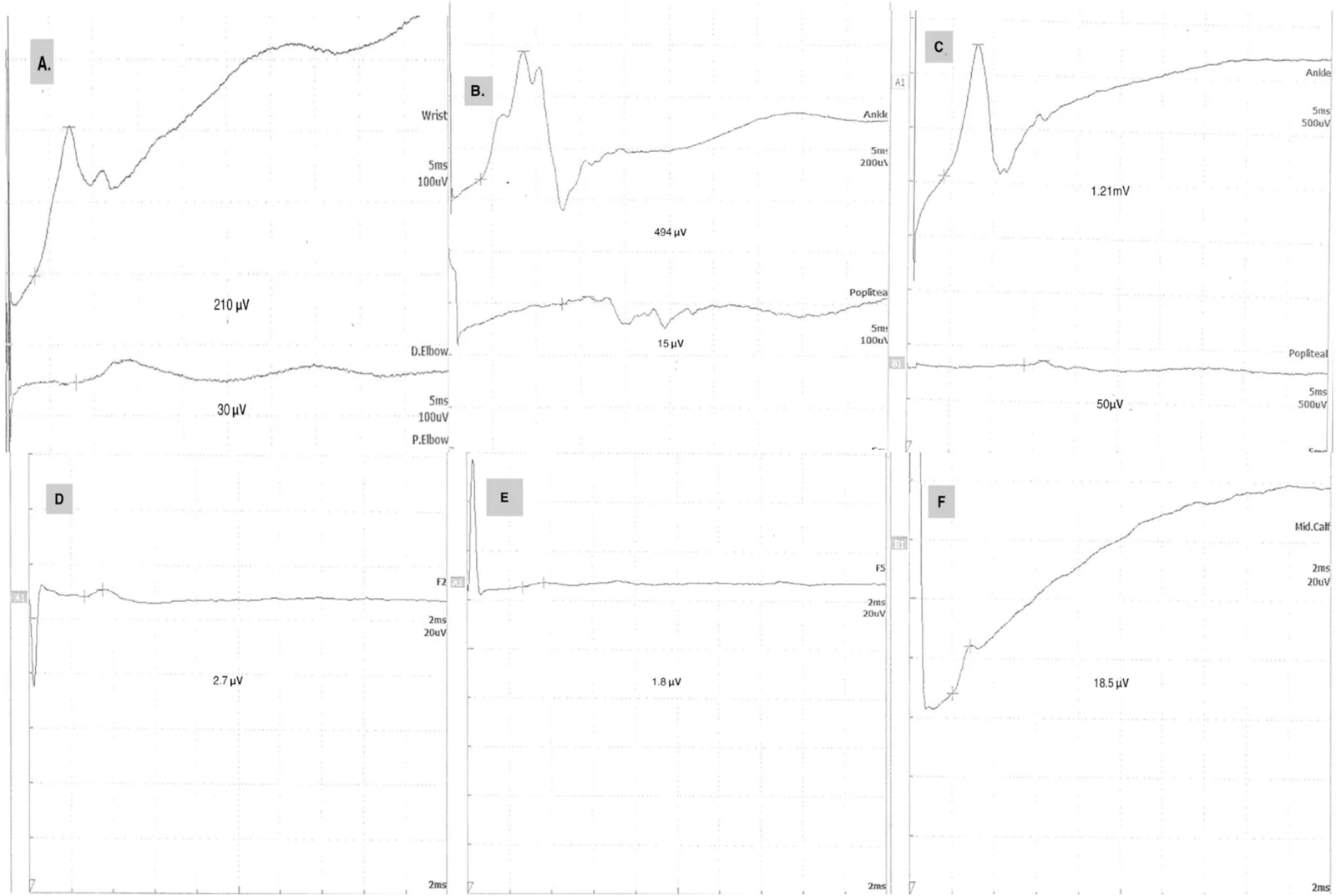
Motor nerve conduction studies demonstrated absent responses in the bilateral median nerves, right ulnar nerve, bilateral radial nerves, and bilateral peroneal (fibular) nerves. (A) the left ulnar compound muscle action potential (CMAP) exhibited reduced amplitude, with evidence of significant conduction block (>85% in the right ulnar nerve across the forearm; (B–C) > 95% in both tibial nerves upon stimulation at the popliteal fossa). Sensory nerve conduction studies revealed markedly reduced amplitudes of the bilateral median and ulnar mixed palmar sensory nerve action potentials (SNAPs) (D–E), with relative preservation of the sural, radial, and superficial peroneal sensory responses bilaterally (F).

Intravenous immunoglobulin (IVIG) was initiated promptly on the first day of hospital admission (one day after symptom onset) at a dose of 0.4 g/kg/day for five consecutive days; however, the patient showed no clinical improvement. Consequently, a brain MRI performed 10 days after symptom onset demonstrated multifocal T2- and FLAIR-hyperintense lesions involving the periventricular, subcortical, and juxta cortical white matter of both cerebral hemispheres. No contrast enhancement was observed on post-gadolinium sequences. Spinal MRI showed longitudinally extensive cord swelling with T2/FLAIR hyperintensity from the medulla to the conus medullaris, with patchy post-gadolinium enhancement (Figure 2).

**Figure 2:**
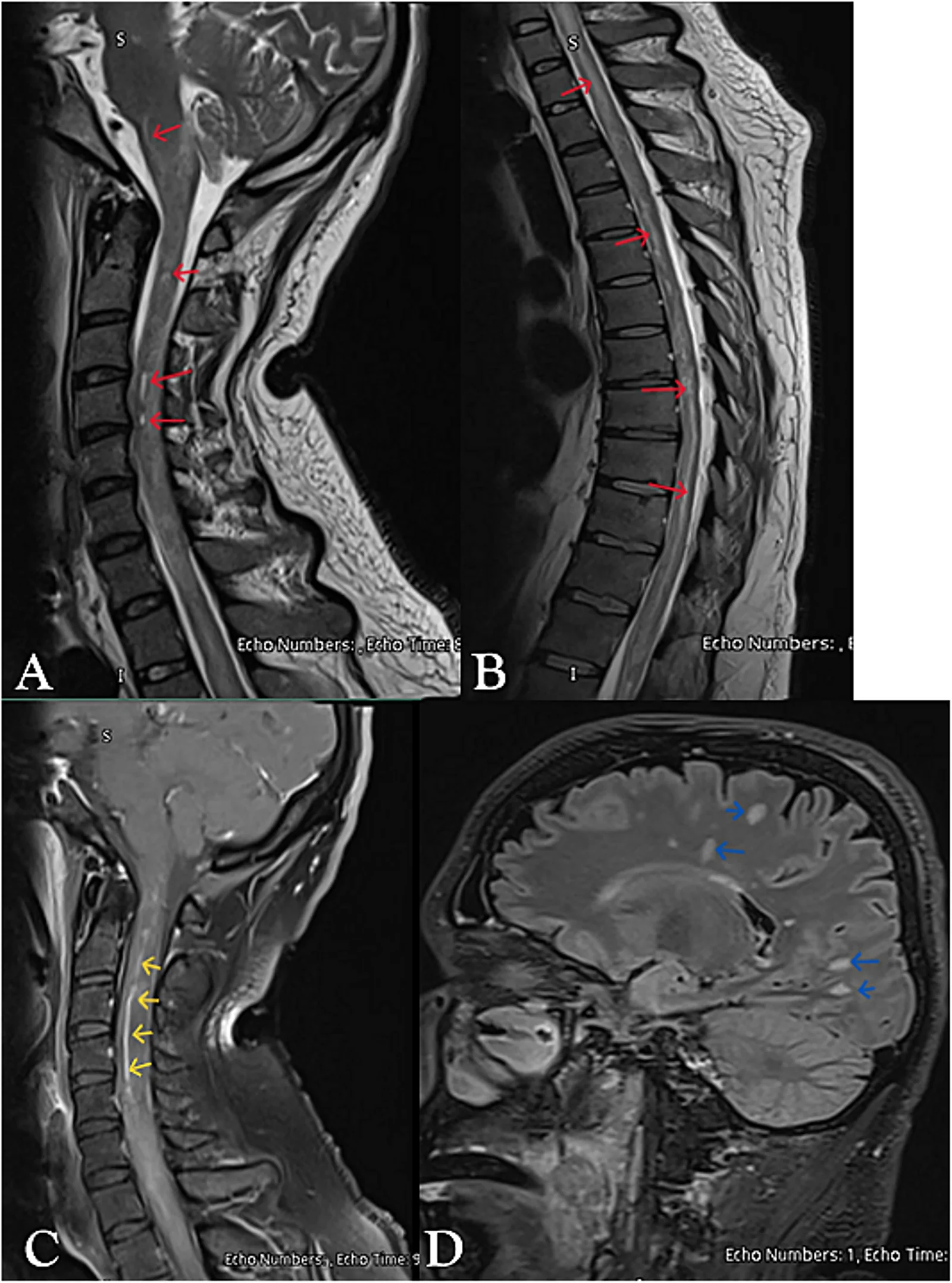
(A–B) sagittal T2-weighted MRI demonstrates a longitudinally extensive T2 hyperintense lesion extending from the pontomedullary junction to the conus medullaris (red arrows). (C) Sagittal post-contrast T1-weighted MRI shows patchy enhancement following gadolinium administration (yellow arrows). (D) Sagittal brain FLAIR shows multiple hyperintense lesions, a few with ovoid morphology and perpendicular orientation to the lateral ventricles (blue arrows).

Subsequently, on the 10th day of hospital admission, plasma exchange therapy was initiated. The patient underwent a total of seven sessions, performed on alternate days, resulting in partial stabilization but without meaningful neurological recovery. High-dose intravenous corticosteroids were administered following imaging evidence of central nervous system demyelination. Due to persistent disease activity, a second course of intravenous immunoglobulin (IVIG) was administered 20 days after completion of the initial course, at a dose of 0.4 g/kg/day for five consecutive days.

During the course of illness, significant autonomic dysfunction emerged, characterized by episodes of profuse sweating, urinary retention, labile blood pressure, and recurrent bradycardia, which progressed to asystole and necessitated emergent intervention.

The patient’s clinical course was further complicated by recurrent episodes of sepsis, which delayed both escalation to advanced immunotherapy and permanent pacemaker implantation. Rituximab was ultimately initiated four months after symptom onset. Despite aggressive multimodal immunotherapy, neurological recovery remained minimal. At six months following symptom onset, the patient had achieved only limited neurological improvement and remained quadriplegic, tracheostomy-dependent, ventilator-dependent, and pacemaker-dependent.

## Discussion

In this case we present a rare clinical presentation of overlapping central and peripheral demyelination. The severe AIDP subtype of GBS that our patient had is characterized by primary demyelinating neuropathy with secondary axonal degeneration and is associated with a more aggressive clinical course compared to pure demyelinating forms of GBS. Patients often present with rapid progression of weakness, early respiratory compromise, and prolonged recovery. In our case, the fulminant progression requiring early mechanical ventilation and the poor initial response to intravenous immunoglobulin (IVIG) are in line with previously described severe AIDP cases. The further need for escalation to plasma exchange also supports the refractory nature of AIDP in such extreme cases.

The main unusual feature of this case is the presence of multifocal CNS demyelination on magnetic resonance imaging, including periventricular and juxta cortical lesions with Dawson finger–like configuration and enhancing spinal cord plaques, which likely explain the patient’s hypoesthesia below the C4 dermatome. Such findings raise a broad differential diagnosis, including acute disseminated encephalomyelitis (ADEM), multiple sclerosis (MS), neuromyelitis optica spectrum disorder (NMOSD), and myelin oligodendrocyte glycoprotein antibody-associated disease (MOGAD). Although the radiological appearance fulfilled criteria for dissemination in space and time, the acute, monophasic clinical course, a clear antecedent infection, and the absence of intrathecal oligoclonal bands (with only matched bands in CSF and serum) argue against classical MS and instead support a post-infectious inflammatory demyelinating process. Furthermore, serum protein electrophoresis with immunofixation showed no monoclonal band, making a paraprotein-associated neuropathy less likely. In addition, the absence of encephalopathy makes classical ADEM less typical, and negative AQP4-IgG and MOG-IgG testing argues against NMOSD and MOGAD, respectively; however, an ADEM-like post-infectious demyelinating phenotype within a broader overlap syndrome remains possible.

The coexistence of acute peripheral neuropathy and CNS demyelination has been increasingly described under the umbrella of combined central and peripheral demyelination (CCPD) [3]. Our case supports this evolving concept, showcasing that immune responses after infections might target shared or overlapping antigens across both systems, leading to mixed clinical presentations of both CNS and PNS involvement. Proposed mechanisms include molecular mimicry triggered by preceding infections, resulting in cross-reactive antibodies against gangliosides and myelin-associated proteins.

Another important aspect of this case is the presence of severe autonomic dysfunction, manifesting as recurrent bradycardia, hypotension, and episodes of circulatory collapse requiring pharmacological support and pacing measures. Autonomic involvement is a recognized complication of GBS, occurring in a significant proportion of severe cases, but can be life-threatening if not promptly managed [4]. The recurrent peri-arrest episodes triggered by minimal stimuli in our patient underscore the need for meticulous cardiovascular monitoring and avoidance of vagal stimulation in such cases. The successful stabilization with isoprenaline infusion and supportive measures aligns with current management strategies for severe dysautonomia in GBS.

From a therapeutic standpoint, this case highlights the importance of a multimodal immunotherapy approach in complex neuroimmunological overlap syndromes. The lack of response to initial IVIG therapy necessitated the escalation to corticosteroids and plasma exchange, reflecting the need for individualized treatment strategies when standard therapy fails. The potential role of second-line immunosuppressive agents, such as rituximab, may be considered in refractory cases, particularly when an underlying antibody-mediated process is suspected.

In conclusion, this case contributes to the growing body of literature describing overlap between central and peripheral demyelinating disorders and highlights the need of considering CNS involvement in patients with severe presentations of GBS. Early recognition, comprehensive neuroimaging, and aggressive multidisciplinary management are essential to improve outcomes in these rare but challenging clinical scenarios.

## Data Availability

Data can be obtained from the corresponding author upon request.
